# The Perceptions and Experiences of Mobile Health Technology by Older People in Guangzhou, China: A Qualitative Study

**DOI:** 10.3389/fpubh.2021.683712

**Published:** 2021-06-25

**Authors:** Jiong Tu, Manxuan Shen, Jiudi Zhong, Gang Yuan, Miaohong Chen

**Affiliations:** ^1^School of Sociology and Anthropology, Sun Yat-sen University, Guangzhou, China; ^2^Department of Geriatrics, The First Affiliated Hospital of Sun Yat-sen University, Guangzhou, China; ^3^Department of Thoracic Surgery, Sun Yat-sen University Cancer Center, Guangzhou, China

**Keywords:** older people, health technology, mobile health services, qualitative research, China

## Abstract

The study explores older people's perceptions and experiences with mobile technology adoption in hospitals. Twenty nine older people were interviewed at a tertiary hospital in Guangzhou from June to December 2020. All the interviews were analyzed using thematic analysis. Older people are a diversified group. Various factors impact their readiness for technology use, including their educational level, age, past experiences, living arrangements, etc. The older people in this study in general expressed a great concern about using the new health technology and many encountered barriers to its successful adoption. Yet, the barriers and difficulties that they encountered are embedded in a changed social context in China. The findings above provide insights into the adoption of health technology, and tailored measures to facilitate older people's technology adoption are suggested.

## Introduction

China entered an aging society after 2000 (1). In 2019, the number of the population over the age of 60 has already surpassed 254 million, reaching 18.1 percent of the whole population (2). Since 2000, China has also rapidly entered the internet age. According to a report by the China Internet Network Information Center (CNNIC), by June 2020, China has 940 million internet users, among whom 97 million were over the age of 60 (3). Although the number of older internet users is rising, still it covers just over 10 percent of all users. This means that at least over 157 million older people use the Internet rarely or not at all. The wide spread of the Internet and Mobile Technology goes hand-in-hand with the aging of the population. What does it mean for older people in an Internet era? Through the case of mobile technology adoption for hospital access, the study explores older people's experience of living in an Internet era.

The setup of internet hospitals and smart hospitals has become a trend in most Chinese public hospitals since 2015 (4, 5). Information technology has been encouraged to be used in medical institutions to provide safe and appropriate services. Besides providing healthcare services online through e-hospitals or internet hospitals (5, 6), now the major hospitals in Chinese cities have also adopted a smart system—the so-called “Internet + medical care” platform—to streamline the hospital procedures, aiding non-clinical procedures, such as appointment registration, and payment in particular (7). The process has sped up during the COVID-19 pandemic, from providing online consultations, prescriptions and drug delivery (8), to moving many hospital procedures online.

Previous studies have demonstrated the various advantages and improvements that Internet and Mobile Technology has brought to the health care sector; for instance, increasing accessibility for populations facing barriers to health care access, especially those in remote and rural areas, improving health care accessibility, efficiency, and the quality of health care, and reducing queuing and waiting time at the hospital (5, 7, 9–11). Yet, mobile technologies also bring challenges related to health care access. Some studies explore the impact of technology on the already overwhelmed health professionals (12), hard-to-reach populations, and the elderly. It suggests that adopting new technology within the healthcare system may unintentionally privilege populations with more resources while exacerbating the disparities for others (13). In general, young people are more likely to use web-based health tools. Older people, with more health issues, tend to use the communication technology the least. A study based in China also finds that a readiness to use e-hospitals is based on technological proficiency, and that young people are more proficient compared to older people (6).

Older people are the most frequent and heaviest users of health services (14). In an aging society and internet age, there is an urgent need to explore how they respond to these new changes in public hospitals. Most of the current researches on older people's use of mobile and digital health technology are quantitative in nature (5, 14–17), which may appear insufficient for understanding older people's perceptions and lived experiences of technology adoption. A qualitative approach would provide a more nuanced understanding of the participants' perspectives, grounded in their specific social contexts (18). Hence, this study adopts a qualitative approach to understand older people's experiences and perceptions of smart technology in hospitals and, in the process, identify the factors that influenced the adoption of new technology by older people. We hope that the results will provide some insights for policy regarding technology adoption and the integration of older people into hospital access.

## Methods

**Figure 1:**
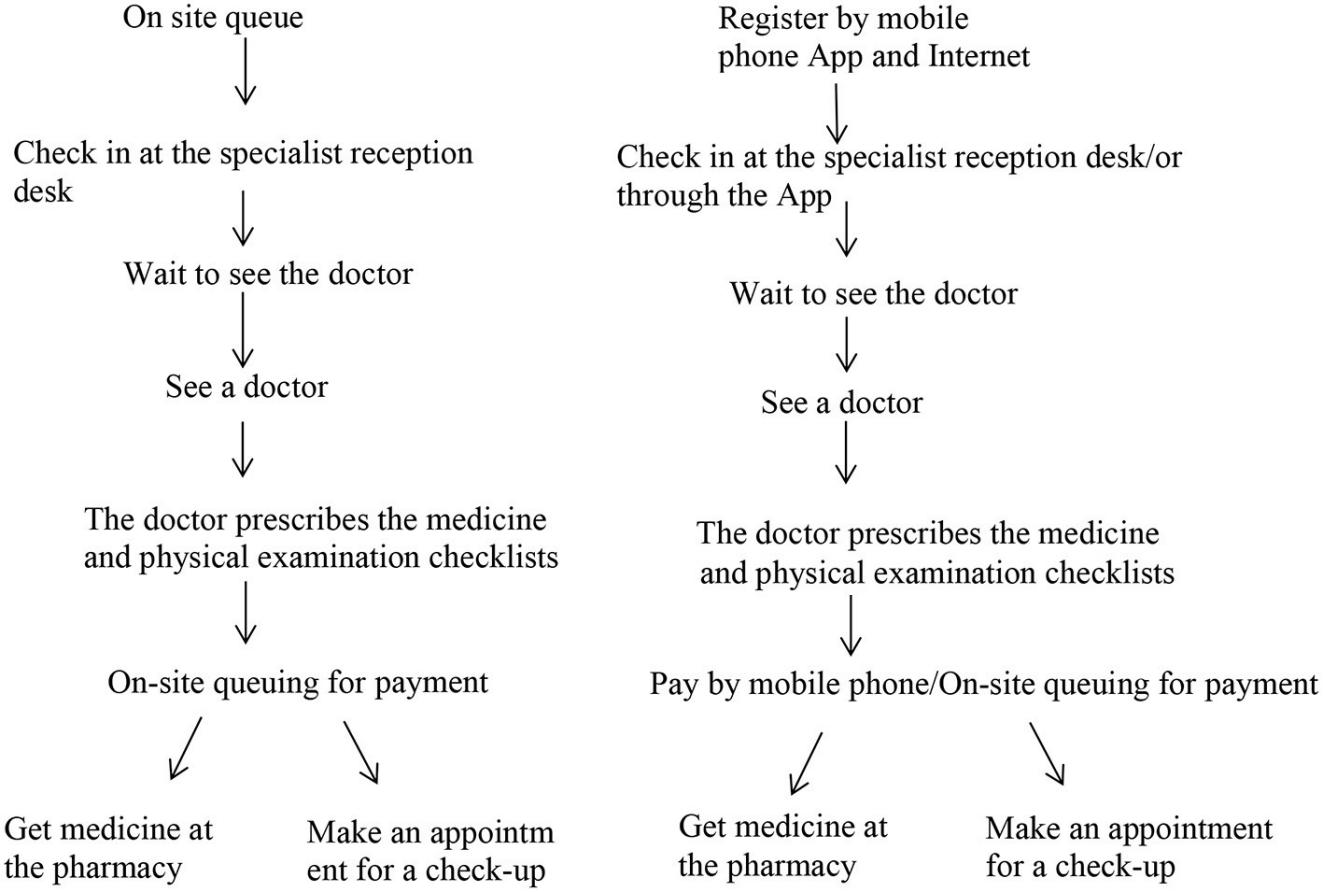
Comparison between the past and the current hospital procedures for outpatient services.

The study was carried out at a tertiary hospital in Guangzhou city, a metropolitan area in southern China. Guangzhou is aging rapidly, with 18.25% of its whole population over the age of 60 by the end of 2018, which is above the country's average (19). The city is pioneering hospital reform, especially with regard to adopting smart technology to improve the hospital procedures. The hospital where the study took place was one of the examples (see Figure 1 below). The health professionals in this hospital frequently heard older patients complaining about hospital access since the adoption of the smart technology.

In order to understand older people's experiences in-depth, we recruited 29 older people to be interviewed when they came to the hospital's geriatric department for health care from June to December 2020. The geriatric clinics were visited by older patients with various conditions. The older people were randomly recruited after their outpatient visit and were interviewed at the side of the clinic. The first two authors, experienced in qualitative study, conducted the interviews. Since the hospital's adoption of the smart technology, all patients were required to make appointment online before hospital visit, hence all interviewees had experienced with the new technology either in person or with the help of others. The participants were asked about their recent hospital experiences and attitudes toward smart technology in the hospital (see Appendix 1 Interview Guide): (1) how do they access hospital services now? Do they encounter any difficulties? How do they solve them? (2) What is their attitude toward new health technology? (3) What should be done to improve the situation? We interviewed to saturation, when we found that the viewpoints and themes that the older people were expressing were repeated and no new points were emerging. The interviews lasted from 20 to 40 min. All of the interviews were audio recorded with the interviewees' consent, then transcribed verbatim. The interviews were then analyzed deductively using thematic analysis (20). The first author and a research assistant coded the data independently to identify the main themes concerning older people's perceptions and experiences of technology. All themes identified were further discussed and scrutinized by the research team to research an agreement. Besides interviewing older people, the research team used the hospital's online system in person, which deepened our understanding of older people's experiences. Albeit carrying out in one hospital, the study with a case study approach was suitable to explore the specific social context regarding a group of selected population. The study was approved by the authors' Institutional Review Board (IRB Approval no. 2019-425).

## Results

As shown in Table 1, the interviewees enrolled in the study were aged between 63 and 87, with a mean age of 73.5. Referring to the retirement age (55 for women and 60 for men in China) and previous study (21), we classified all of the interviewees into three groups in Table 1 below: the young old (aged 60–69 years); the middle old (aged 70–79 years) and the very old (80+). All of the interviewees were retired. The majority of them had a high school and above educational degree, representing a relatively higher educated group among older people who have a relatively low education level in general (22). There were more females than males.

**Table 1:** Characteristics of the older people interviewed (*n* =29).

**Characteristics**	***N* (%)**
**Age, year**	
60–69	11 (37.9)
70–79	10 (34.5)
≥80	8 (27.6)
**Gender**	
Male	9 (31)
Female	20 (69)
**Educational level**	
Junior high school and below	4 (13.8)
Senior high school	8 (27.6)
College or above	17 (58.6)

### Comparison of the Past and the Current Hospital Procedures

The hospital started to move some service procedures online, such as appointment bookings, hospital guidance, payment, and report checking in 2019 (see Figure 1). People can carry out these procedures either through the hospital's mobile App or through the hospital's account on Wechat (a popular social application in China). On these web-based platforms, the hospital also provides a wide range of health information and knowledge to the public.

For a regular hospital visit, it is a complex process with many procedures: making an appointment, registration, waiting to see the doctor, consulting the doctor, payment, the physical examination, obtaining medication, etc. Tertiary hospitals in the urban cities were often overcrowded with patients, making every procedure even longer: long registration and long waiting and queue times, while the consultation time with the doctor was relatively short. The older people whom we interviewed had much to say about the traditional procedures.

*You needed to queue for registration, queue to see the doctor, queue for payment, and queue at the (hospital) pharmacy to obtain medicine; if the doctor prescribed both Chinese and western medicine, you needed to queue twice (for they were located in two different pharmacies). Normally, for an outpatient visit, it would take me three to four hours, a whole morning…*. (Interviewee1, 75 years, male).

The adoption of the smart system by many hospitals aimed to simplify the hospital procedures, in the process improving the accessibility of health services for patients. The convenience services provided through the online platform include intelligent guidance (hospital procedure, maps, etc.), registration (online booking of an appointment with a medical expert), mobile payment, inspection report query, medical feedback, health education, etc. From Figure 1, we can see that, for an outpatient visit, the online platform saved patients several rounds of queuing from the appointment registration, checking in to payment. If more procedures (for instance, physical check-ups) are needed for an outpatient visit, it would save patients more time if they used the mobile App in terms of inspection report checking. Many older people experienced the changes that the smart system brought.

*In the past, there was no online system. We had to come on site very early to queue for an outpatient registration ticket. I used to get up at 4 am, came to the hospital at around 5 am, and queued till 7 am when the registration office opened. Now I don't need to worry (very much) about registration, (my adult child helps me) to make an appointment online and I come to the hospital at around the appointment time*. (Interviewee4, 79 years, male)

*It is convenient (now), I do not need to queue (for registration). In the past, I came here to queue at 5 am in the morning. Now I come at around the time of my appointment, and there is no crowd of people at the entrance (queuing) anymore*. (Interviewee10, 75 years, female)

### Different Technology Experiences for Older People With Diversified Backgrounds

Older people have varied experiences of using the Internet and Mobile Technology. According to our interviewees, most of them use mainly the leisure and social functions of their smartphone (e.g., using Wechat to chat, making video/phone calls, watching videos, and reading the news), and some also use mobile payment. A few of the interviewees rarely or never use a smartphone or computer.

In terms of mobile healthcare access, different users demonstrate quite different attitudes and experiences. Among the 29 interviewees, 16 interviewees received help from family or friends to access online healthcare services (for instance, registering for a hospital appointment). Thirteen of the interviewees carried out the online appointment registration themselves, but sometime encountered difficulties. One (Interviewee10, 75 years old, female) even taught others how to use it, because she was a teacher at a local university and used computers quite often in her daily life, which constituted an unusual case among older people in the same age group. The ones who used the mobile healthcare system themselves tended to have a higher educational/literacy level, and used computers quite often in their past work.

*I do not have a problem (with the online system), but why is it difficult for many older people? For me, I used computers quite often in my (past) work, so now the (hospital) mobile App is similar to what I dealt with in my work. But, for most other people, it would be difficult*. (Interviewee1, 75 years, male)

*Other (older people), who saw I could make an appointment on the phone, always expressed their admiration: ‘Oh, you can register on the phone.' I always told (myself to) learn as early as possible, or else it would be impossible when I got dementia… It is convenient to register online, but only a small proportion (of older people) can handle it, most elderly cannot, it becomes a trouble then. I know how to do it, because I used to be an English teacher in middle school and typed (on a computer) often*. (Interviewee14, 77 years, female)

Factors such as technology literacy, education level, past working experiences, and age all impact on older people's technology adoption. Yet, the low literacy level among the older generation itself constrains many people's ability to adopt smart technology (22). Some of the older people whom we interviewed expressed a sense of uncertainty and even panic about visiting a hospital alone since the new change. It is not an environment and a procedure with which they are familiar.

*I feel we need to have second thoughts about the digitalization of hospital procedures, because different people have different ways of adapting to it. Young people may adapt quickly, but older people may not understand (the change). Like me, I saw young people typing on my phone quickly (helped me to register), but I did not understand at all. People have different experiences of it*. (Interviewee11, 83 years, female)

### Conflicting Views: “It's Convenient, but…”

All older people in this study realized that online appointment/registration for outpatient visits was replacing the traditional channel for registration. Most of the older people expressed a seemly conflicting view; while admitting the advantages and convenience that the smart system brought, they also pointed out the problems and challenges that older people encountered. Even the ones who knew how to use the online system admitted that, if all procedures move online in the future, older people would be excluded.

*It is quite convenient now, you can register online. If your schedule changes, you can cancel the appointment (in advance). You don't need to go to the hospital, don't need to queue (on site). But sometimes I don't know (how to use it)… Because I am alone, my daughter doesn't care about me, I have to explore everything by myself, or else I go to the hospital to ask the girls (receptionists) at the entrance to teach me…* (Interviewee16, 72 years, female)

*For those who know how to use (the App), it becomes more convenient. But for us older people, sometimes we don't know how to operate that, so it becomes a real trouble… each time I had to ask help from health professionals. I know how to use the App to make an appointment, but all other things I am not familiar with. Each time a slight change (in the hospital procedure) becomes a difficulty for older people. As long as we do not know how to use it, the new change means more complexity for us* (Interviewee11, 83 years, female)

*You need to register an account first, set the password, fill in all kinds of (electronic) forms… The system also is updated now and then, it then changes. We would like a more fixed (system)*. (Interviewee18, 71 years, female)

To make an appointment using the hospital online system, the first step is to create an account, whereby users need to register their information (name, age, gender, ID card number, etc.) online and link their ID card with their hospital account. The process seems too complex for many older people. Moreover, the App changes or upgrades frequently, so the older people indicated that they needed constantly to learn and often encountered difficulties. While admitting the convenience that technology brought, some interviewees expressed a sense of uncertainty about facing a changing system and pointed out their preference for the fixed, old procedure. Most of the older people whom we interviewed just use the online system to manage their appointments, while completing all other procedures offline. In these cases, the new technology did not fundamentally change the complex hospital procedures.

*I still pay by cash, queuing in front of the cashier's window. The change is good. But no matter how convenient it is, as long as I can't use it, it becomes inconvenient* (Interviewee4, 79 years, male).

*I have been seeing doctors in the hospital since my 30s. Actually, it should be more convenient, now the procedure to see a doctor has been simplified a lot. But, because of our situation, the key is that we cannot catch up with the progress of society, and it becomes difficult. For those who know how to use a mobile phone, it's really easy. But we are passive (in the process), always waiting for others' help, I feel it is difficult* (Interview19, 79 years, female).

### Uncertainty and Confusion: “Society Is Hard for Us Older People”

For the older patients who were unfamiliar with or did not use the mobile App to manage their health access, the new change in the hospital means not an improvement or simplification, but a more complex and difficult process. The digital platform presented new uncertainties for the older people. The online registration quota ran out quickly. Sometimes, the older people were unable to get an appointment online and still relied on offline channels by asking the doctors to give them an extra registration, which however would depend on whether the doctors have the time for outpatient consultations.

*I asked my daughter to stay online waiting for a new appointment (with the doctor I always saw), yet we could hardly succeed one time out of ten tries. We don't know how the system operates and when new registrations are available online*. (Interviewee2, 65 years, female)

*I've no idea how to use it. It's always been my sons who helped me with the registration. In the past, I did everything all by myself. Now it has become very complex, I'm afraid to use it, can't deal with it … In the past, it was not that complicated. Now, too complex, I still can't accept it*. (Interviewee7, 74, female)

Many older people are unfamiliar with new technology in general and health technology in particular. When encountering difficulties that they had not experienced previously, they became agitated and angry: “Society is hard for us older people,” “We (older people) are miserable,” “Society has become advanced, but we're left behind” many proclaimed in the interviews. From their point of view, older people seem unable to stop the progress of society, and have a low level of confidence about living in a technology-dominated society. They are uncertain about the result of using new technology, scared to make mistakes, and prefer a fixed, familiar procedure.

*My son taught me twice. He also helped me set up the Wechat payment and Alipay. But I sometime typed the wrong password (for the payment), or repeated the payment. Sometimes, I have to change to cash. We are lagging behind; others do not pay with cash anymore*. (Interview5, 65 years, female)

*I hope things could be like in the past; don't change the procedures, if they can. Or leave an old channel for us, even it means I come very early and queue for a long time… I still queue to pay by cash. (Is it troublesome?) No, at long as it allows me to (pay). At worst, it's a little slow, but I can see the doctor and obtain my medication. Regarding the online (procedure), I know nothing; what could I expect?* (Interviewee23, 81 years, male)

For some of the interviewees, low confidence in facing new technology resulted in a natural rejection of it. In comparison to the futility and anxiety of using mobile health technology, some older people preferred to use the traditional offline procedure for booking hospital visits, with which they were familiar. At least, it enabled them to get the needed health care by themselves, albeit with long queues and waiting times.

### An Added Sense of Dependence, Burden, and Futility: “I Daren't Go to the Hospital Without My Daughter, but She's Busy…”

With the adoption of new technology in hospitals, older people rely more and more on others for hospital access: adult children, grandchildren and other family members. Living with (adult) children becomes an essential factor that may impact on older people's hospital access. It is then difficult for those without a family to help. There are decreasing numbers of older people living with their children. If no family members are around, older people may ask for the help of various friends, neighbors, hospital staff, or even employ somebody to help them attend the hospital.

*We two (a couple) live by ourselves; we don't know how to use it. Each time we have to find (employ) a (care worker) to help us (make an appointment). All of our information is on his mobile phone*. (Interviewee6, male, 87)

*Some rely on adult children and even grandchildren for help but, for those living alone, there are no kids around to help. Some had only a nanny helping at home, but the nannies from rural areas may be unfamiliar with the hospital's (web-based) system. Some had to seek help from outside the family*. (Interview17, 64 years, female)

Relying on other's help reduces the older people's sense of independence and self-efficacy. In their interviews, some older people expressed a sense of burden and guilt. They are frequent hospital visitors, yet have to ask for their younger family members' help every time. They feel sorry about troubling others and dissatisfied about having to rely on others for assistance, while in the past they could handle all of the hospital procedures themselves.

*I feel troubled (by the new change). I don't know how to use it and have to ask a favor from others*. (Interviewee20, 77 years, female)

*If my son helped me, I feel I troubled him, because he's very busy and has a lot of pressure (at work)*. (Interviewee5, 65years, female)

*We older people are indeed unfamiliar with those things on a mobile phone, and how to use it. It's better if there are young people at home to help. But if no young people are at home, what could we do? Now, we are like disabled people, no longer useful*. (Interviewee15, 84 years, female)

When asking for help, the older people feel that they are burdening their younger family members. Even when visiting the hospital by themselves, they have to rely on the hospital staff or other younger patients' help for some hospital procedures. Several older people even cannot attend the hospital without being accompanied by their younger family members, which deepens their sense of dependence and futility.

### Attitudes Toward Learning New Technology

Many older people do not want to rely on others, but if there were an opportunity for them to learn how to use the mobile technology, how would they react? Of the 29 interviewees, only 10 expressed a willingness to learn how to use the new technology. The reason why they wanted to learn was mainly to become independent and avoid troubling others, because they are frequent hospital visitors and health care access is a necessity.

*Now, we're growing older and older, so seeing a doctor will become more frequent. We can't always ask the kids for help. They have work to do, and are frequently away on business; they don't have time to always stick around. If there were an opportunity to learn the whole procedure in the hospital, I would love to, and don't want to be asking for help from young people often. But, at the hospital, there is no place and chance for me to learn. They (the staff) would help me to make appointment on the phone, but they operate quickly; I don't know how they do it. As long as my physical condition allows, I'd love to learn*. (Interviewee27, 69 years, female)

None of the others expressed a clear willingness to learn, including eight people who clearly rejected the idea of learning how to use the new technology. The older people, including those who wished to learn, pointed out various difficulties that they faced, which are listed in Table 2.

**Table 2:** Perceived barriers to learning and using mobile health technology.

**Barriers**	**Explanation**
1. Physical condition	Poor eyesight Poor memory Reduced manual dexterity: difficulty typing on a mobile phone Poor health status: physical condition makes learning impossible
2. Devices	No smartphone Unfamiliar with or rarely use electronic devices (smartphones or computers) No Wi-Fi connection
3. Learning difficulties	Low literacy level Cognitive capabilities: unable to understand (quickly); declining problem-solving ability No one to teach patiently (young family members unavailable, hospital staff too busy) Hospital online system too complex, App not elder-friendly
4. Other factors	No time to learn: family obligations take up most of their time Low confidence, self-conceived inability

The older people encountered many difficulties in learning and using smartphones and the App. Although there is an increased prevalence of electronic devices, many older people still use cheap, older phones or not so smart smartphones. Some again need support from the younger generation to purchase and use a smartphone. Many of the older people have a low level of information technology skills, often learn slowly and need someone to teach and guide them with great patience. The hospital did have volunteers and staff at the reception to guide people on how to gain hospital access, yet these few volunteers could hardly meet the needs of a large numbers of patients. The older people even compared their experiences of using mobile phones in banks and hospitals, as they had someone carefully guide them on how to use a mobile phone to transfer money at the bank, but did not get the same guidance at the hospital.

*I would love to learn (how to operate the hospital online system), but my memory is poor. If I ask the young people many times, they become annoyed. I need to find a notebook, write down in detail what should I tap first, what to do next…I have to practice many times (in order to remember)*. (Interviewee2, 65 years, female)

*I do not have a willingness to learn. I'm old, my memory is poor, people told me several times (how to register), but I still can't remember. If there are young people to help (me), I will let them … If young people are at my side, teaching patiently, perhaps I could, but they're too busy, have their own work to do. Besides, my mobile cannot get online*. (Interview4, 79 years, male)

The older people's attitudes toward learning new technology were impacted by their self-perceived ability. The aging process is linked to declining physical and cognitive capabilities, which influence their self-perceived ability to learn.

*We can't (use the App) because of memory issues. If I learn now, I'll forget a few days later if I don't use it… I'm slow to get online, I don't know how to use the computer … To learn, there are two concerns. One is my physical condition, if it's OK, I can attend. The other is time. I'm still taking care of (grand) kids, need to take and collect the kids from school every day. So only if the time is in between can I attend (the training programme)*. (Interviewee19, 79 years, female)

*They can't even use a (smart) mobile phone. I often touched the screen and went to a wrong section, but did not know how to exit… I just had an operation for cataracts, now my eye's OK. But, for many older people, they cannot watch the screen clearly. It's tough … I feel a cashier window (to obtain a registration number) is necessary. Or else, what should we do? If every older person brings their mobile phone, asking the lady (receptionist) for help, there's just one lady there to respond, so older people will queue for even longer*. (Interviewee12, 63 years, female)

The above listed barriers make some older people scared of learning, feel low in confidence and hold a low self-perception of their ability to learn. During the interview, many of the older people expressed a strong sense of futility regarding the new technology and suggested leaving in place a traditional offline channel for booking a visit to the doctor for older people who are unable to learn.

## Discussion

The study explores older people's experiences and perceptions regarding the use of the Internet and Mobile Technology to gain hospital access, in the process identifying the major barriers and potential facilitators for older people.

### Difficult Experiences and Complex Feelings

Internet and Mobile Technologies are being adopted by Chinese hospitals to streamline and simplify the hospital procedures. Yet, the new online platform makes hospital service access technology-dependent. Many of the interviewees admitted that the new technology made hospital access convenient, but only for those who could use a mobile phone and the hospital App.

Older people are a diversified group, with varied ages, educational levels, working experiences, prior experiences with information technology, information technology skills, and family circumstances, all of which impact on their attitudes and adoption of health technology. As we found in our study, older people, particularly those who are younger with a high educational level and those who used electric devices in their past job, tend to have higher technology literacy, a greater willingness to adopt health technology to book hospital visits. The findings were consistent with other studies, that revealed that socio-demographic factors influence older people's acceptance and adoption of health technology; for instance, young-older people with a higher education, higher income, wider social network, and good health status were more likely to report health information technology use (13, 15), while those who belong to the older age groups, with a low income and low education level, are significantly less likely to use Web-based health care services (16).

Different from the previous studies, however, the older people in this study (including those who could use the hospital App relatively easily) generally expressed a great concern regarding the adoption of mobile technology by the hospital. Those with limited technology experience encountered great difficulties related to successfully adopting technology at some stage. Health problems and healthcare access difficulties increased with age (23), and the adoption of smart technology by hospitals seemed to increase the difficulties related to accessing healthcare for some of the older people, if they did not receive timely help from younger people.

The experiences of using health technology also have emotional consequences for older people. Their unfamiliarity with new technology, low level of confidence about using electronic devices, uncertainty about the result of using the hospital App, and fear of making mistakes all give older people a sense of uncertainty and frustration. Moreover, the new change in hospitals forced many older people to rely on younger people, family members in particular, for help with daily hospital access. This is similar to the situation in other countries, where older adults need family support to use of health technology devices (24), yet the family members tend to “do” instead of “teach” (25). Besides, the young family members may not always be available. This added to the older people's sense of dependence and being a burden, and some stated that they felt useless and left-behind by the progress of society. Some demonstrated resistance toward the new technology and still preferred to use the offline channel for health care, and a similar attitude has been found among older people in other countries (26).

The older people's willingness to learn also varied. The reasons why many older people did not want to learn are diverse, including their physical condition (poor health, poor eyesight, manual dexterity, etc.), availability of devices (no smartphone, no Wi-Fi connection), learning difficulties (poor learning ability, no people to teach, low confidence, etc.) and so on. Our finding on the barriers to technology adoption is cognate with existing studies about the difficulties related to seniors' adoption of health information technology (6, 17, 24). Yet, what we found is a more complex picture of various factors that contribute to older people's difficulties, and these factors are related to the changing family structure and the problems existing in the healthcare system as a whole. Older people living with the younger generation have a higher usage of e-hospitals (6). While Chinese family culture benefits many seniors to get help from their family members, the changing family structure (reduced family size due to the one-child policy) and household arrangements (increase in the number of elderly “empty nesters” who live alone) may negatively impact on older people's technology adoption. In terms of the healthcare system issue, while adopting smart technology, the hospital did not match it with sufficient aid for older people. Besides, the hospital APP in this study is reported by many older people as difficulty to use. Elder-friendly App designs should consider common age-related changes in vision, hearing, cognition, memory, problem solving ability, and manual dexterity (27). Yet, many health applications may not suit older adults to use in China (28). Finally, why did many older people choose to visit the top level hospital, where health technology was first piloted to streamline its procedure? In an ideal hierarchical healthcare system (as promoted by the current health reform), community and primary medical institutions should have responsibility for chronic disease management and refer patients with serious conditions to major hospitals; however, in practice, these institutions were short of medicine and advanced medical equipment, with low use and capacity of primary healthcare providers (29). Many older people, albeit with non-urgent chronic conditions, still rely on tertiary hospitals for health care.

### Policy Implication

The policy design should consider the diversified experiences and general problems that older people encountered and introduce measures targeted at assisting older people with technology use and hospital access.

First, it is important to provide more technical guidance and support for older people regarding hospital access. Older people are slower to adapt to new technologies. Difficulties and a fear of learning also arise because no one is around to teach them patiently and there is no clear guidance offered at the hospital. Training might be provided for the elderly who have the willingness and ability (capacity) to learn. This training can happen within the hospital as well as the community. Within the hospital, clearer guidance and more timely assistance from the staff or volunteers should be arranged when a new system is introduced. In terms of the community, this is where older people are easily accessible and digital health coaching programs can be carried out by the community and social work institutions.

Second, it is essential to develop elder-friendly and secure Apps and devices. The hospital APP older people use in this study was updated regularly and constantly changing. The procedures to use the APP were unclear even for the older people with greater technological capacity. Besides, different hospitals have different systems, and users have to learn and adapt to a different system each time they attend a new hospital. It adds to the sense of complexity for elderly users. Overall, the App design should consider people with all kinds of literacy levels and offer user-friendly interfaces to facilitate older people's use.

Third, it is important to leave in place a traditional channel for those unable to learn or use mobile technology. Many older people indicate that they prefer a fixed hospital procedure. Almost all of the interviewees emphasized that it was essential to leave open an offline channel for older people, especially those with a low literacy level and who were unwilling or unable to learn. Besides, the healthcare system should be better organized to strengthen the community-based services, where older people with non-urgent chronic conditions can easily access the needed health services, instead of visiting the major hospitals.

## Limitations

As indicated above, older people are a diversified group with different backgrounds and educational levels. The people we interviewed were from a capital city in a major metropolitan area in China, seeking health care at a top level hospital. The older people in these regions may have a higher educational level and greater technological literacy compared to those living in the remote and rural areas. Hence, the study, being based at a top level hospital, may not represent the experience of older people in other medical institutions and regions across the country. Besides, since all of the participants were recruited within the hospital after their outpatient visit, they were the ones who had successfully obtained high-quality hospital services. The ones who were willing to participate in the interview were generally more able to express themselves (cognitively) and had a better health condition. Hence, they may also represent the ones with a better chance to use the technology.

Despite these limitations, the study involving these relatively advantaged older people already exposed many difficulties and challenges related to adopting the Internet and mobile health technologies. It could be predicted that the situation is even worse for other older people and that more urgent measures should be taken to involve older people more effectively in using public services. Further studies may consider examining the diversified experiences of older people in other regions.

## Conclusions

The study explores older people's perceptions and experiences regarding mobile technology adoption in hospitals. Older people are a diversified group. Various factors impact their readiness for technology use, including their educational level, age, past experiences, living arrangements, etc. While experiencing the convenience of new technology, older people in generally express great concern and many encountered barriers to successful adoption. The new online platform makes hospital service access technology-dependent. It forces many older people to rely on others to arrange hospital visits for them, which adds to their sense of burden and futility. The findings above provide insights into the adoption of new technology. Overall, collaborative efforts involving family members, communities, hospitals, and the government are needed to make older people in general enjoy the progress of society.

## Data Availability Statement

The original contributions presented in the study are included in the article/Supplementary Material, further inquiries can be directed to the corresponding authors.

## Ethics Statement

The studies involving human participants were reviewed and approved by ICE for Clinical Research and Animal Trials of the First Affiliated Hospital of Sun Yat-sen University. The patients/participants provided their written informed consent to participate in this study.

## Author Contributions

JT wrote the manuscript. MS, JZ, GY, and MC revised the manuscript. All authors contributed to the data collection and analysis.

## Conflict of Interest

The authors declare that the research was conducted in the absence of any commercial or financial relationships that could be construed as a potential conflict of interest.
