# Supplementary material for: The Perceptions and Experiences of Mobile Health Technology by Older People in Guangzhou, China: A Qualitative Study
Source: Front Public Health. 2021 Jun 25;9:683712. doi: 10.3389/fpubh.2021.683712 (PMC8267812; doi:10.3389/fpubh.2021.683712)
Supplement: Supplementary file 1 [file Table_1.DOCX]

## Appendix 1

**Interview Guide**

1. How do you register for hospital services? How do you feel?
2. Is there anyone to help you with registration or appointments? If so what help do they provide?
3. Do you know how can you access hospital registration now? What do you know?
4. Except for registration, for other hospital procedures, have you used online services? How do you feel about that?
5. Have you visited this hospital previously? What was the hospital procedure like in the past (a few years ago)? How do you feel about it compared to the current procedure?
6. Are you willing to learn how to use the online services (e.g., online registration, online payment)? What difficulties might you encounter? Do you have any suggestions?
7. What would you think if the hospital were to move all non-clinical services online? What suggestions do you have?
8. Do you use electronic devices (e.g., mobile phones) in daily life? What kind of devices do you use? What do you use them (it) for?
