# Supplementary material for: The Perceptions and Experiences of Mobile Health Technology by Older People in Guangzhou, China: A Qualitative Study
Source: Front Public Health. 2021 Jun 25;9:683712. doi: 10.3389/fpubh.2021.683712 (PMC8267812; doi:10.3389/fpubh.2021.683712)
Supplement: Supplementary file 2 [file Table_3.DOCX]

**Supplementary material:**

**Table A: Consolidated criteria for reporting qualitative studies (COREQ) checklist.**

| **Domain 1 : Research team and reflexivity** | |
| --- | --- |
| **Personal characteristics** |  |
| 1. Interviewer | **Jiong Tu; Manxuan Shen** |
| 2. Credentials | PhD, RN, MD |
| 3. Occupation | 1 Medical Sociologist, 3 nursing researcher, 1 Doctor |
| 4. Gender | 4 female, 1 male |
| 5. Experience & training | Teaching qualitative methods, training in qualitative research methods |
| **Relationship with participants** | |
| 6. Relationship established prior to  study commencement | No |
| 7. Participant knowledge of the  interviewer | Yes, reasons for doing research |
| 8. Interviewer characteristics | Reported in method part of the main document |
| **Domain 2: Study design** | |
| **Theoretical framework** |  |
| 9. Methodological orientation & theory | Qualitative study based in one institution, case study approach, thematic analysis |
| **Participant selection** |  |
| 10. Sampling | Random sampling |
| 11. Method of approach | Face to face interview |
| 12. Sample size | 29 in total |
| 13. Non-participation | Some old people did not have time or very sick and unable to participate after the outpatient visit |
| 14. Setting of data collection | At the side of the clinic after older patient’s outpatient visit |
| 15. Presence of non-participants | No |
| 16. Description of sample | Outlined in the main document (previously in the methods part, now in the result part) |
| **Data collection** |  |
| 17. Interview guide | Questions draft, piloted and revised |
| 18. Repeat interviews | No repeat interviews |
| 19. Audio/visual recording | Interviews were audio-recorded |
| 20. Field notes | Recorded after interviews |
| 21. Duration | Reported, range 20 to 40 minutes |
| 22. Data saturation | Sampling continued until data saturation |
| 23. Transcripts returned | Transcripts were available to participants on request |
| **Domain 3: analysis and findings** | |
| **Data analysis** |  |
| 24. Number of data coders | Outlined in the text, two in total |
| 25. Description of coding tree | A coding tree was not developed |
| 26. Derivation of themes | Themes were derived from the data by thematic analysis |
| 27. Software | No, coding by hand in the word file |
| 28. Participant checking | No, the older people came to the hospitals were changing daily, unable to meet the same patients during the research period. |
| **Reporting** |  |
| 29. Quotations presented | Supporting quotations presented |
| 30. Data and findings consistent | Yes |
| 31. Clarity of major themes | A clear presentation of major themes is outlined |
| 32. Clarity of minor themes | Variations in views and themes and minor themes are  presented |
